# Novel combinations to improve hematopoiesis in myelodysplastic syndrome

**DOI:** 10.1186/s13287-020-01647-1

**Published:** 2020-03-20

**Authors:** Khaja Syed, Sara Naguib, Zhao-Jun Liu, Luisa Cimmino, Feng-Chun Yang

**Affiliations:** 1 University of Miami Miller School of Medicine, Miami, FL 33136 USA; 2 Janssen Research and Development, Malvern, PA 19477 USA; 3 UT Health San Antonio, San Antonio, TX 78229 USA

**Keywords:** Myelodysplastic syndrome, Myeloid-derived suppressor cells, Transfusion independent, T regulatory cells, Leukemic stem cells, Therapeutic antibodies

## Abstract

Myelodysplastic syndrome (MDS) represents a heterogeneous group of clonal hematopoietic disorders, which is characterized by cytopenias in the peripheral blood and bone marrow dysplasia due to ineffective hematopoiesis. Patients with MDS have an increased risk of transformation to acute myeloid leukemia (AML). Although the molecular basis of MDS is heterogeneous, several studies demonstrated the significant contribution of the dysregulated immune system in accelerating MDS progression. The immunosuppressive tumor microenvironment is shown to induce tolerance of MDS blasts, which may result in a further accumulation of genetic aberrations and lead to the disease progression. Increasing evidence shows an expansion of myeloid-derived suppressor cells (MDSCs), a population of inflammation-associated immature cells, in patients with MDS. Interestingly, the increased MDSC populations are shown to be correlated with a risk of disease progression in MDS. In addition, MDS is highly prevalent in aged individuals with non-hematology co-morbidities who are fragile for chemotherapy. Increasing research effort is devoting to identify novel agents to specific targeting of the MDSC population for MDS treatment.

## Introduction

Myelodysplastic syndrome (MDS) is a group of clonal hematologic disorders characterized by the abnormal and ineffective hematopoiesis with an increased risk of acute myeloid leukemia (AML) transformation [1]. Patients with MDS usually present constitutional and debilitating symptoms, including fatigue, fever, and severe unusual and recurrent infections [2, 3]. The key features in the bone marrow (BM) of MDS patients include impaired functions of hematopoietic stem and progenitor cells, dysregulated differentiation of myeloid, erythroid, and megakaryocytic lineages, as well as dysplastic hematopoietic cells. Recurring genetic mutations involving in histone modification, DNA methylation, transcription factors, RNA splicing, DNA repair, cohesion complex proteins, kinase signaling, and several signal transduction elements have been identified to contribute to the pathogenesis of MDS [4, 5]. Cytogenetic abnormalities have also been shown to influence the clonal architecture and may provoke an inflammatory BM microenvironment to promote clonal expansion, thus promoting the development of MDS. MDS is known as age-related stem cell disorder impacting elders greater than 65 years. Although the majority of cases have an undefined etiology, it has been shown that the determinant factors leading to MDS include exposure to chemicals, chemotherapy, and high doses of radiation [6, 7].

As a standard prognostic tool, in MDS patients, International Prognostic Scoring System (IPSS) is the clinical and pathological assessment of morphology, periphery cytopenias, and karyotype [3, 8], which predict the transformation of MDS to AML. MDS patients can be categorized into four groups, including lower-risk, intermediate 1, intermediate 2, and high-risk MDS. Based on the IPSS scoring system, the prognostic subgroups differ significantly in the rates of survival and leukemic transformation [9, 10]. MDS patients of low and intermediate 1 risk groups have a longer overall survival rate than the patients who are in intermediate 2 and high-risk MDS groups. Interestingly, MDS patients who are at the low and intermediate 1 risk are often accompanied by myelosuppression with resultant cytopenia and anemia. Conversely, patients with high-risk MDS can rapidly transform into AML with a short median survival rate, generally in months [11, 12].

Clinically, the management for MDS is often complex due to the age, disease stage, and co-morbidities of individual patients. The treatment options for MDS patients range from supportive care to aggressive treatment, such as chemotherapy and hematopoietic stem cell transplantation [13, 14]. MDS patients with IPSS low or intermediate 1 risk MDS are typically offered with supportive care, such as red blood cell transfusion, cytokine, and antibiotics to prevent infection [15, 16]. The lower-risk MDS patients with the absence of chromosomal del 5q aberration can be treated with erythropoiesis-stimulating agents (ESAs) or other growth factors specific for hematopoiesis [17, 18]. High-dose ESAs, combined with G-CSF, have yielded erythroid response rates in this setting in the range of 30 to 50% and of median duration 2 years [19–21]. In contrast, patients who are in intermediate 2 or high risk generally require prompt treatment, such as chemotherapy or a stem cell transplantation [22–24]. Decitabine and azacytidine (AZA) are nucleosides and act, in part, by incorporating into DNA as false cytosine residues that cannot be methylated by DNA methyltransferase and form covalent adducts with the enzyme [25–27] This interaction leads to the depletion of DNA methyltransferase to reverse the aberrant methylation that silences key genes with the tumor-suppressive activity [28, 29]. AZA treatment has exhibited improved overall survival for MDS patients who are at high-risk [30]. Most patients treated with a hypomethylating agent do not achieve an objective response. Combinatorial treatment with AZA and Revlimid demonstrated synergistic effects in MDS owing to targeting of different pathways [31, 32].

While the molecular basis of MDS is heterogeneous, increasing evidence revealed the significant contribution of the dysregulated immune system in accelerating MDS progression [33]. The immunosuppressive tumor microenvironment is shown to induce tolerance of MDS blasts, which may result in a further accumulation of genetic aberrations and lead to the disease progression. Several groups reported that MDS patients have an expansion of myeloid-derived suppressor cells (MDSCs), a population of inflammation-associated immature cells. Interestingly, the increased MDSC population is associated with a risk of MDS progression [33]. In this review, we summarized the current understanding of the involvement of immunosuppressive tumor microenvironment in MDS initiation, progression, and potential treatment.

## Innate immune system in MDS

In the tumor microenvironment, the primary immunosuppressive cell types include activated immature myeloid cells, Tregs, and regulatory B cells. Upon activation, these immunosuppressive cells reduce the T cell proliferation and type II interferon secretion, inhibit antigen presentation, and inhibit natural killer cell function [34]. MDSCs secrete high levels of soluble factors with inflammatory suppressive activity, such as sCD27, sCXCL8, sCSIF and transforming growth factor beta. Tregs modulate self-tolerance and immune surveillance, which inhibit the effective immune responses against the malignant clone and accelerate disease progression. In addition, the regulatory B cells suppress T cell proliferation by modulating the production of IL-10 and altering cellular contacts. A higher frequency of immunosuppressive cell populations has been reported in the peripheral blood and the BM of the patients with MDS compared to that of healthy individuals. It has also been shown that the immunosuppressive cell populations can alter normal hematopoiesis through a direct contact with stem cell and progenitor cells, which may contribute to anemia development [34]. Thus, targeting these immunosuppressive cell populations may be beneficial to patients with anemia and thus reduce frequent RBC transfusions and promote myeloid maturation, benefiting MDS patients [34].

**Fig. 1:**
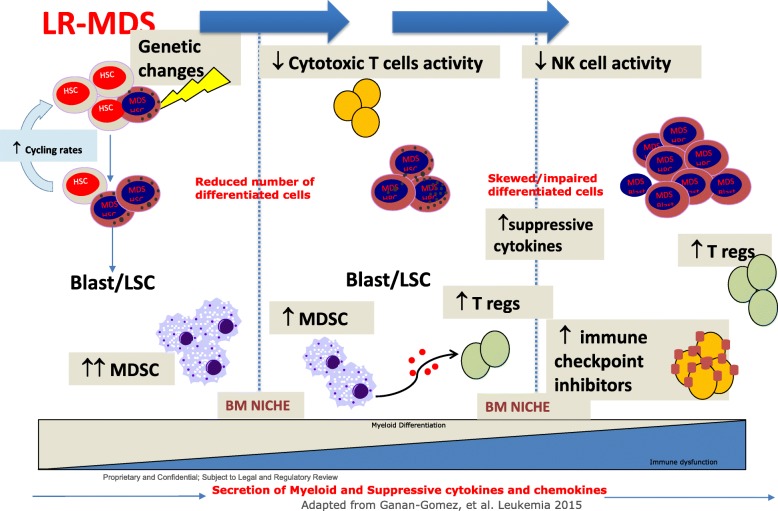
Toxic chemical exposure, genetic changes, or aging lead to the generation of malignant clone in the bone marrow, which affects the normal hematopoiesis. This early dysregulation in hematopoietic system leads to reduction of mature cells in the peripheral blood. Within the MDS bone marrow microenvironment, the immature cells and MDSCs secrete immunosuppressive cytokines to reduce effector T cell proliferation and contribute significantly to the dysregulation of immune surveillance in MDS. LR-MDS (low-risk MDS)

Below as illustrated in Fig. 1, increased populations of inflammatory cells in tumor microenvironment are a prominent feature of MDS, which canresult in a suppression of normal hematopoietic cell differentiation [34]. In the last decade, clinical investigations and studies in mouse model systems revealed that MDSCs, a heterogeneous group of cells originated from myeloid lineage, were drastically expanded in the BM of MDS patients, which may contribute to the pathogenetic development of ineffective hematopoiesis, and thus can be used as an indicator for poor prognosis of MDS patients. Surprisingly, MDSCs in MDS patients do not acquire the same somatic gene mutations as the MDS clone, suggesting that these MDSCs may arise from a distinct hematopoietic clone rather than the MDS clone. Regardless of the potential impact of MDSCs on the MDS pathogenesis, the mechanisms of action remain to be investigated. MDSCs secrete immunosuppressive cytokines to reduce effector T cell proliferation and contribute significantly to the dysregulation of immune surveillance in MDS and possess strong immunosuppressive activities, potentially through secreting immunosuppressive cytokines and interacting with other immune cell lineages, including T cells, macrophages, dendritic cells, and natural killer cells [34].

Phenotypic studies on human MDSCs revealed that MDSCs lack the traditional surface markers of mature immune cells, including lineage negative (LIN^−^), HLA-DR^−^ [35]. However, MDSCs express CD33, CD123, and CD38, and CD27L is expressed on MDSCs [36, 37]. CD33 is a sialic acid-binding lectin usually expressed on immature myeloid cells and in the myeloid lineage that is not expressed on hematopoietic stem/progenitor cells [38]. The high frequency of CD33 cell population in the bone marrow of MDS patients signifies the involvement of its impact on the disease initiation and progression [38].

CD123 is a transmembrane glycoprotein capable of binding to IL-3 receptor beta (CD131) and can form a complex that can signal through the cell membrane [5, 39]. Two isoforms of CD123 have been reported which are capable of binding IL-3 which leads to malignant cell survival by a signal through the cell membrane [40, 41]. The shorter isoform, which is missing a portion of the extracellular domain close to the N-terminus, generally expressed at significantly lower levels than the longer isoform [41]. CD123 is the marker on leukemic stem cells (LSC), and the high frequency of this antigen on LSCs is shown to correlate to blast proliferation and poor prognosis [42]. CD123 has been shown to be expressed with high levels of Lin^−^ HLA-DR^low^ CD11b^+^ CD33^+^ MDSCs, an increase in the frequency of MDSCs in MDS patient samples compared to healthy controls [43].

CD27, a costimulatory receptor of the TNF superfamily, is constitutively expressed on lymphocytes and hematopoietic stem/progenitor cells [44]. We found that CD27L is expressed on Lin^−^ HLA-DR^low^ CD11b^+^ CD33^+^ MDSCs and the frequency of Lin^−^ HLA-DR^low^CD11b^+^CD33^+^CD27L^+^ MDSCs is much higher in the BM than that in the peripheral blood of MDS patients [45].

CD38 is a glycoprotein with ectoenzymatic functions, which is expressed on different cells, including lymphocytes and myeloid cell populations. We have preliminary data showing that MDS patient has a higher frequency of CD38^+^ cell population on immune-suppressive MDSC [46].

### Antigen-specific therapeutic antibodies targeting immune suppressive cells in the tumor microenvironment of patients with MDS

The fact that MDSCs function as a key cellular component of the BM microenvironment of MDS patients points to a potential role of MDSCs in the initiation and progression of MDS. It is conceivable to hypothesize that inhibition of the MDSC population may benefit to MDS patients. In fact, several studies have revealed the potential of depleting MDSCs or inhibiting MDSC activity in the treatment of MDS patients.

Gleason and colleagues developed a single-chain variable fragment (scFv) recombinant reagent, so-called bispecific BiKE, which targets CD16 as well as the myeloid differentiation antigen CD33 (CD16xCD33) and facilitates CD33^+^ cell elimination [38]. They further showed that CD16xCD33 BiKE can reverse MDSC immunosuppression of NK cells and induce MDSC target cell lysis [38]. This study implies a potential of using the CD16xCD33 BiKE to target MDSCs in MDS patients. In another study, to improve hematopoiesis in MDS patients, a therapeutic drug developed with Fc-engineered CD33 monoclonal antibody has been tested to target MDSC [35], blast, and leukemic stem cell population in patients with low-risk MDS and preventing immune-suppressive cytokine secretion by blocking CD33 antigen with the goal of improving bone marrow hematopoiesis [47].

Anti-CD123 is a humanized IgG1 monoclonal antibody that specifically targets the α-subunit of the IL-3 receptor (CD123) to effectively neutralize IL-3 signaling [48]. This therapeutic antibody has been engineered to have increased affinity for FcγRIIIa (CD16), which elicits potent, receptor density-dependent killing, thus eliminating target cells via [1] antibody-dependent cellular cytotoxicity and [2] macrophage-mediated antibody-dependent cellular phagocytosis, respectively. This drug is also shown to be able to eliminate cells expressing elevated levels of CD123, such as basophils, plasmacytoid dendritic cells, myeloid-derived suppressor cells, leukemic blasts, and leukemic stem cells. Of note, elimination of CD123^+^ MDS blasts, leukemic stem cells, and MDSCs thus offers a new therapeutic strategy for AML patients [49].

Daratumumab is a human IgG1ĸ monoclonal antibody (mAb) that binds with high affinity to a unique epitope on CD38, a transmembrane glycoprotein. It is a targeted immunotherapy directed towards tumor cells expressing high levels of CD38, such as plasma cells from patients with multiple myeloma. Daratumumab is shown to eliminate CD38^+^ immunosuppressive cell populations, including MDSCs, Tregs, and Bregs [46]. Due to notable efficacy in heavily pretreated multiple myeloma patients, daratumumab was approved by both the FDA (2015) and the EMA (2016) as mono-therapy to treat relapsed multiple myeloma.

## Conclusion

Treatment of patients with MDS has improved in recent years, but remains challenging. Drugs targeting different pathways or different cell populations might show a better overall response in MDS to minimize side effects. Synergistic effect by targeting tumor cells and immunosuppressive populations shows a promising result in improving bone marrow hematopoiesis. Combinatory application of ezatiostat hydrochloride and epoetin alpha in MDS patients at lower risk may be able to reduce the red blood cell transfusion dependence. Combination therapy using hypomethylating agents or Revlimid together with an antigen-specific therapeutic antibody would increase the response rates and improve patient quality of life with minimal adverse side effects.

## Data Availability

Not applicable
